# Associations between obesity, smoking behaviors, reproductive traits and spontaneous abortion: a univariable and multivariable Mendelian randomization study

**DOI:** 10.3389/fendo.2023.1193995

**Published:** 2023-07-20

**Authors:** Qingyi Wang, Fanglei Liu, Yinfeng Tuo, Li Ma, Xiaoling Feng

**Affiliations:** ^1^ Department of First Clinical Medical College, Heilongjiang University of Chinese Medicine, Harbin, China; ^2^ Department of Gynecology, The First Affiliated Hospital of Heilongjiang University of Chinese Medicine, Harbin, China

**Keywords:** spontaneous abortion (SA), Mendelian randomization (MR), smoking behaviors, obesity, reproductive traits

## Abstract

**Background:**

The correlation between potential risk factors such as obesity (leg fat percentage (left), arm fat percentage (left), waist circumference, body fat percentage, trunk fat percentage), smoking behaviors (past tobacco smoking, smoking initiation, smoking/smokers in household, current tobacco smoking) and reproductive traits (age first had sexual intercourse (AFS), age at menarche (AAM), and age at first birth (AFB)) have been linked to the occurrence of spontaneous abortion (SA). However, the causal associations between these factors and SA remain unclear.

**Methods:**

We conducted univariable and multivariable Mendelian randomization (MR) analyses to evaluate the associations of obesity, smoking behavior and reproductive traits with SA. To select appropriate genetic instruments, we considered those that had reached the genome-wide significance level (P < 5 × 10^–8^) in their corresponding genome-wide association studies (GWAS) involving a large number of individuals (ranging from 29,346 to 1,232,091). SA was obtained from the FinnGen consortium, which provided summary-level data for 15,073 SA cases and 135,962 non-cases.

**Results:**

Assessed individually using MR, the odds ratios (ORs) of SA were 0.728 (P = 4.3608×10^-8^), 1.063 (P = 0.0321), 0.926 (P = 9.4205×10^-4^), 1.141 (P = 7.9882×10^-3^), 5.154 (P = 0.0420), 1.220 (P = 0.0350), 1.228 (P = 0.0117), 0.795 (P = 0.0056), 1.126 (P = 0.0318), for one standard deviation (SD) increase in AFS, AAM, AFB, smoking initiation, smoking/smokers in household, arm fat percentage (left), leg fat percentage (left), waist circumference and body fat percentage, 0.925 (P = 0.4158) and 1.075 (P = 0.1479) for one SD increase in past tobacco smoking, trunk fat percentage for one SD increase in SA. In multivariable MR (MVMR), only AFS (OR = 0.802; P = 0.0250), smoking initiation (OR = 1.472, P = 0.0258), waist circumference (OR = 0.813, P = 0.0220) and leg fat percentage (left) (OR = 4.446, P = 0.043) retained a robust effect.

**Conclusion:**

Smoking behaviors, reproductive traits and obesity-related anthropometric indicators are potential causal factors for SA. Higher leg fat percentage; smoking initiation; and lower waist circumference and AFS may increase the risk of SA. Understanding the causal relationship for SA may provide more information for SA intervention and prevention strategies.

## Introduction

Spontaneous abortion (SA) usually refers to the loss of a fetus before a specific gestational week. It primarily includes an empty gestational sac, the gradual cessation of embryonic development, the death of the embryo or fetus, the expulsion of the embryo and its appendages, etc. In the case of SA, pregnancy varies from 20 weeks to 28 weeks (1–4). According to statistics, Females of reproductive age have a 10% chance of experiencing SA (5). If they are not promptly intervened with, they will not only impose a severe economic burden on the patient and her family, but also exert a substantial influence on the physical and mental well-being of individuals.

The correlation between potential risk factors such as obesity, smoking behaviors, and reproductive traits and the occurrence of SA has been a major focus of epidemiological research. However, the findings from numerous observational studies have not been consistent, indicating the possibility of variations in research designs, population samples, measurement methods, and statistical analysis. Furthermore, the presence of reverse causality, misclassification, unobserved confounding, and other biases often hinder the determination of a causal link between these risk factors and SA and only provide partial evidence of a correlation. This uncertainty makes determining which risk variables genuinely contribute to the occurrence of SA and how to properly incorporate them into preventative and treatment methods more difficult. Therefore, to gain a better understanding of the risk factors associated with SA, it is crucial to employ more accurate and reliable methods for studying the relationship between these factors and the occurrence of SA. Mendelian randomization (MR) designs, as an emerging causal inference method, have the potential to overcome the limitations of observational studies and provide a more precise evaluation of the causal link between risk factors and SA. In a bid to minimize the effect of residual confounding, MR designs use genetic variants as instrumental variables (IVs) to assess causality. The presence of reverse causality and confounding is highly unlikely due to the random distribution of genetic variants during pregnancy, which remains unaffected by environmental or self-adopted lifestyle confounding factors. Consequently, MR designs provide more reliable and accurate evidence. Studying the risk factors of SA through MR designs can better guide the development of effective prevention and treatment strategies.

Consequently, to investigate the potential causative relationship of obesity (leg fat percentage (left), arm fat percentage (left), waist circumference, body fat percentage, trunk fat percentage), smoking behaviors (past tobacco smoking, smoking initiation, smoking/smokers in household, current tobacco smoking) and reproductive traits (age first had sexual intercourse (AFS), age at menarche (AAM), and age at first birth (AFB)) with risk of SA, we carried out a two-sample univariable and multivariable MR investigation.

## Materials and methods

### Study design and data sources

**Figure 1:**
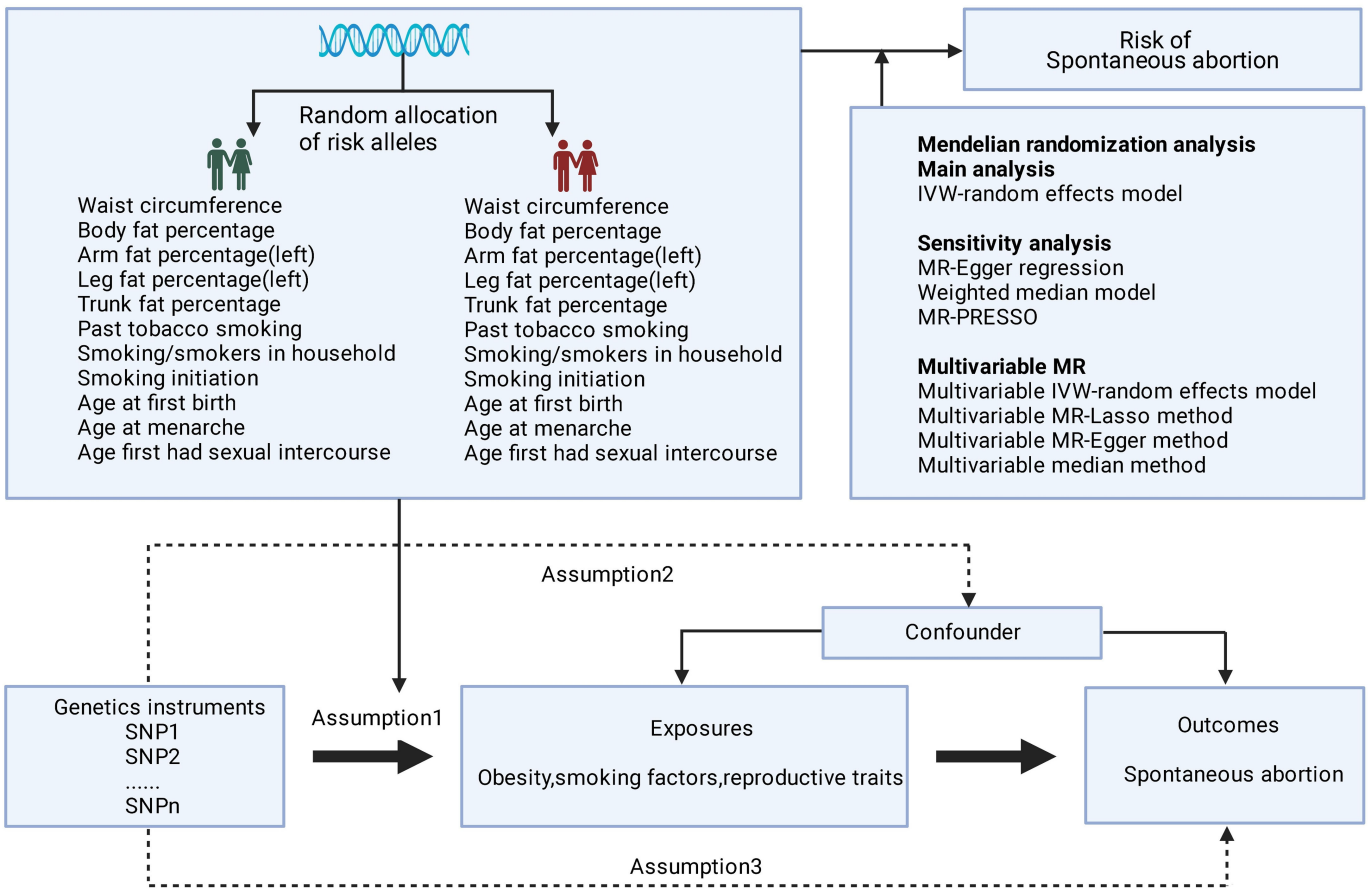
Basic assumptions of Mendelian randomization. The green human symbol indicates lower exposure and the red human symbol indicates higher exposure.

The MR relies on three core assumptions: (1) The genetic variants are strongly associated with the exposure of interest; (2) The genetic variants are independent of any confounders of the exposure-outcome relationship; and (3) The genetic variants affect the outcome only through the exposure, without any pleiotropic effects (**Figure 1**). The genetic instruments utilized in this analysis for the exposures were collected from previously published genome-wide association studies (GWAS). The data for the outcome were obtained from the FinnGen consortium R8 version. SA was defined according to the International Classification of Diseases (ICD) 8th to 10th codes. SA was defined according to ICD codes, as follows: ICD-10 O03, ICD-9 634, and ICD-8 643. The sample size of this study was 151,035, including 15,073 cases and 135,962 controls, all of whom were of white European ancestry. A detailed description of the data sources used in the study is provided in **Table S1**. All studies used in the MR analysis had undergone ethical review and were approved by relevant ethical review boards and participants had provided informed consent prior to their data being used in the analysis.

### Genetic instrument selection

We selected single nucleotide polymorphisms (SNPs) that were associated with leg fat percentage (left), arm fat percentage (left), waist circumference, body fat percentage, and trunk fat percentage at the genome-wide significance threshold (P < 5 × 10^–8^). For smoking behaviors, three sets of instruments SNPs for past tobacco smoking, smoking initiation (whether an individual had ever smoked regularly), and smoking/smokers in household were employed for validation. For reproductive traits, three sets of instruments (SNPs for AFS, AAM, and AFB) were employed for validation.

First, the linkage disequilibrium (LD) between SNPs was calculated for each risk factor using an LD reference panel from 1000 Genomes of European populations. SNPs in LD (r^2 ^> 0.001 and clump window < 10,000 kb) were excluded, and the SNP with the lowest P-value was retained. Palindromic SNPs were defined as those with ambiguous minor allele frequencies falling within the range of > 0.45 and < 0.55 and were subsequently excluded from the dataset (6, 7). A small number of missing instruments in the outcome datasets were not replaced by proxy SNPs, as their limited impact on the results was deemed acceptable. The proportion of phenotype variance explained by the SNPs and the F-statistic was calculated (8). An F-statistic threshold of 10 or greater was deemed appropriate to ensure that the genetic instruments used in the MR analysis were sufficiently strong to meet the assumptions of the method. Additional information on the genetic instruments used in the study is provided in **Tables S2**-**3**. These rigorous data preparation methods are important to ensure the validity and reliability of the MR analysis.

### Main statistical analyses

The primary analyses used inverse variance weighting (IVW) approaches. IVW can combine the Wald ratios of each SNP to produce an unbiased estimate of the causal effect, assuming no horizontal pleiotropy or balanced pleiotropy. To assign weights to the Wald ratios, the IVW method uses their inverse variances. Smaller variances correspond to larger weights, while larger variances correspond to smaller weights. This accounts for differences in precision across the Wald ratios, resulting in a weighted average that serves as an estimate of the causal effect. The study used this method to investigate the relationship between genetic variants and the outcome of interest in the MR study (7).

### Sensitivity analyses

We applied four additional sensitivity analysis methods to examine the robustness and consistency of the results: weighted median, MR-Egger regression, weighted mode, and MR-PRESSO (Pleiotropy Residual Sum and Outlier) (9–11). The weighted median model provides reliable estimations if at least 50% of the weight in the analysis is derived from valid IVs (12). The MR-Egger regression method is useful in identifying and correcting issues associated with directional pleiotropy. However, this approach may result in a trade-off in statistical power. To detect the presence of directional pleiotropy, we employed the P value associated with the MR-Egger intercept term. The MR-PRESSO method is useful in identifying anomalous data points and generating revised estimations after their removal. The MR-PRESSO distortion test is specifically designed to evaluate discrepancies between the initial and revised estimations resulting from the exclusion of identified outliers (13).

We assessed the presence of heterogeneity among the SNPs by computing Cochrane’s Q statistic. Additionally, a “leave-one-out” sensitivity analysis was conducted to evaluate the plausibility of the causal associations and identify any influential SNPs that may affect the robustness of the causal estimations. Furthermore, an analysis of linkage disequilibrium score regression (LDSC) was employed to evaluate the coinheritance of both the exposure and outcome variables.

### Multivariable MR

We conducted a multivariable MR (MVMR) analysis to investigate the direct causal impact of obesity, smoking behaviors, and reproductive traits on the occurrence of SA. MVMR allows for the detection of the causal effects of multiple risk factors on SA simultaneously, while also determining the independent associations of each risk exposure with the outcome. To perform the analysis, we utilized four different MVMR methods, including the multivariable MR-IVW, the multivariable MR least absolute shrinkage and selection operator (LASSO), the Multivariable MR-Egger method, and the multivariable median method. The “TwoSampleMR”, “MR-PRESSO”, “Mendelian Randomization”, and “MVMR” packages in R version 4.2.2 were employed for the analyses. To determine statistical significance, we used a significance level of P < 0.05. Based on our findings, we provide insights into the association between obesity, smoking behaviors, reproductive traits and the occurrence of SA.

## Result

### GWAS of obesity, smoking behaviors and reproductive traits

**Figure 2:**
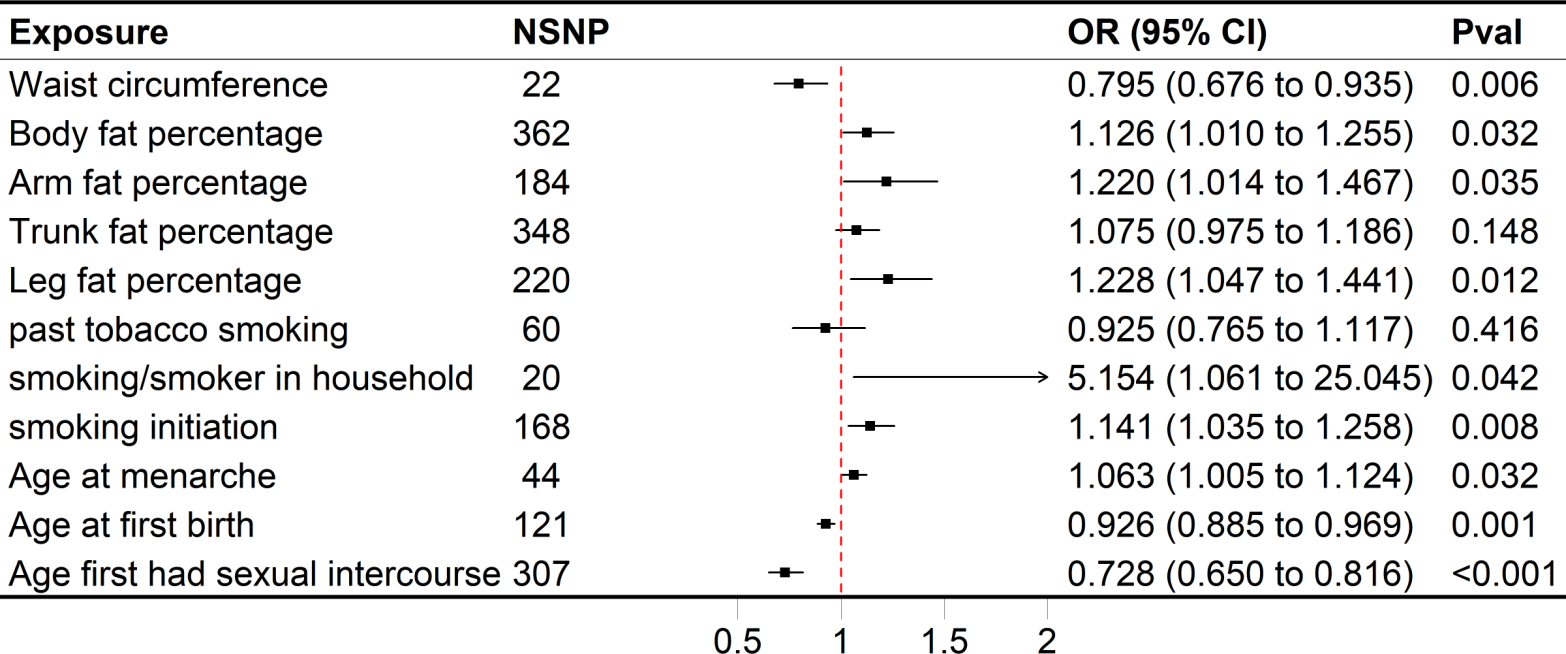
Forest plot of univariable MR results of obesity, smoking behaviors, reproductive traits and risk of SA. Univariable MR estimates were derived using the inverse variance weighted approach. SNP, single nucleotide polymorphism; CI, confidence interval; OR, odds radio.

In the GWAS, we identified a significant number of independent SNPs linked to following traits (P < 5×10^−8^): 220 SNPs were linked to the leg fat percentage (left), 184 SNPs to the arm fat percentage (left), 22 SNPs to the waist circumference, 362 SNPs to the body fat percentage, 348 SNPs to the trunk fat percentage and 60 SNPs for past tobacco smoking (**Figure 2**). We identified numerous independent SNPs with P < 5×10^−6^ linked to following traits, including 168 SNPs for smoking initiation, 20 SNPs for smoking/smokers in household, 307 SNPs for AFS, 44 SNPs for AAM, and 121 SNPs for AFB, respectively (**Figure 2**).

The range of SNPs was from 20 to 348, with the corresponding explained variances varying between 0.013% and 3.535%. Except for smoking/smokers in household IV’s F statistics which are lower than threshold 10, and the other IV’s F statistics are higher than threshold 10, indicating that the evidence of weak instrument bias in this study is low.

### Univariable MR analysis of the association between obesity, smoking behaviors and reproductive traits and SA risk

The associations between variables calculated using a variety of methods (the weighted median, MR-Egger regression, and weighted mode) showed consistent directionality. The results of the four sensitivity analysis methods are shown in **Table S4** and **Table S5**.

Genetically predicted higher arm fat percentage (left), leg fat percentage (left), body fat percentage, and lower waist circumference were associated with a raised risk of SA (**Figure 2**). The ORs of SA were 1.220 (95% CI 1.014–1.467, P = 0.0350), 1.228 (95% CI 1.047–1.441, P = 0.0117), 1.126 (95% CI 1.010–1.255, P = 0.0318) and 0.795 (95% CI 0.676–0.935, P = 5.5636×10^-3^) for arm fat percentage (left), leg fat percentage (left), body fat percentage and waist circumference, respectively. In terms of smoking behaviors, the ORs of SA were 1.141 (95% CI 1.035–1.258, P = 7.9882 × 10^−3^) and 5.154 (95% CI 1.061–25.045, P = 0.0420) for one standard deviation (SD) increase in smoking initiation and smoking/smokers in household, respectively (**Figure 2**). Genetic predisposition to past tobacco smoking and trunk fat percentage (OR = 0.925, 95% CI 0.765–1.117, P = 0.4158; OR = 1.075, 95% CI 0.975–1.186, P = 0.1479) was not associated with SA (**Figure 2**). As for reproductive traits, genetically predicted higher AAM could increase the risk of SA, whereas lower AFS and AFB might elevate the risk of it (**Figure 2**). The odds of SA would increase per 1-SD increase of AAM (OR = 1.063, 95% CI 1.005–1.124, P = 0.0321). Moreover, a 1-SD increase of AFS could help reduce the risk of SA (OR = 0.728, 95% CI 0.650–0.816, P = 4.3608 × 10^−8^), together with AFB (OR = 0.926, 95% CI 0.885–0.969, P = 9.4205 × 10^−4^).

### Multivariable MR analysis of the association between obesity, smoking behaviors and reproductive traits and SA risk

**Figure 3:**
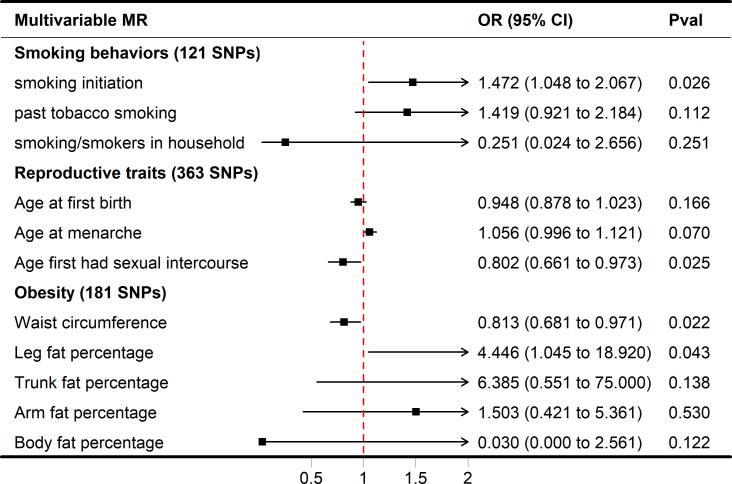
Forest plot of multivariable MR results of obesity, smoking behaviors, reproductive traits and risk of SA. Multivariable MR estimates were derived using the IVW-MVMR.

In MVMR analysis, the relationship between AFS, AAM, AFB, and SA was also evaluated, and only AFS (adjusted OR = 0.802, 95% CI 0.661-0.973, P = 0.0250) had a strong potential causal relationship with SA (**Figure 3**). In the case of mutual adjustment, the point estimates of AFB and AAM were reversed, and the adjusted ORs were 0.948 (95% CI 0.878-1.023, P = 0.1669) and 1.056 (95% CI 0.996-1.121, P = 0.0700). In MVMR–Egger sensitivity analyses, no horizontal pleiotropy was found, and the estimated effect (AFS, adjusted OR = 0.748, 95% CI 0.579-0.968, P = 0.0270) was similar to those observed with IVW-MVMR.

While smoking/smokers in household and past tobacco smoking were not significant in the multivariate MR model (adjusted OR = 0.251, 1.419, P = 0.2507, 0.1122), increased smoking initiation may be an independent risk factor for SA (adjusted OR = 1.472, P = 0.0258). In IVW-MVMR, when examining the genetic relationship between obesity and SA, leg fat percentage was favorably connected with SA (OR = 4.446, 95% CI 1.045–18.920, P = 0.0430), whereas waist circumference was concerned primarily with SA (OR = 0.813, 95% CI 0.681–0.971, P = 0.0220) (**Figure 3**). These findings display a degree of conformity with those observed in the univariable MR (UVMR). The MVMR-Egger sensitivity analysis demonstrated a lack of pleiotropy, indicating very similar impact estimates (AFS, OR = 0.748, 95% CI 0.579-0.968, P = 0.0270; smoking initiation, OR = 1.686, 95% CI 1.106-2.569, P = 0.0150; waist circumference, OR = 0.849, 95% CI 0.669-1.077, P = 0.1770; leg fat percentage, OR = 4.298, 95% CI 1.005-18.392, P = 0.049) when compared to IVW-MVMR (**Figure S1**).

## Discussion

Current MR studies support that smoking behaviors are independently and causally associated with SA risk (14). In addition, our results provide support for the causal relationship between obesity, smoking behaviors and reproductive traits and SA risk. It has been observed that there may be a causal relationship between high leg fat percentage (left) and smoking initiation and increased SA risk, and low waist circumference and AFS are associated with increased SA risk.

SA is one of the most common complications during pregnancy. Its incidence may be affected by many factors, including obesity, smoking behaviors, and reproductive traits. Obesity is a well-studied risk factor for SA and is associated with lower implantation, pregnancy, live birth rates, and slower embryo development (15–22). Female obesity and 6-day trophoblast biopsies have been linked to lower live birth rates in studies (23). Waist circumference and trunk fat percentage are the main indicators for assessing central obesity, while body fat percentage is the main indicator for assessing overall obesity. Body fat percentage refers to the proportion of body fat weight to the total weight, which can be used to evaluate the overall obesity level of individuals. In addition, leg fat percentage and arm fat percentage can provide information about the fat content in other parts of the body. The exact mechanism of this association is unclear, but it is believed to be involved in increased oxidative stress (24), systemic inflammation (25), and shortened telomere length (26). The maternal-fetal interface, which is the site of nutrient exchange and circulation between the placenta and the growing fetus, has been implicated in miscarriage and preeclampsia. A dysfunctional maternal-fetal interface induces oxidative stress in the placenta and the subsequent loss of placental synthetic trophoblast cells, which contributes to the pathogenesis of abortion and eclampsia (27). A study has shown that the normal progression of pregnancy relies on the dynamic balance between oxidases and antioxidant enzymes, and any disruption in this balance leads to pathological complications such as SA (28).

From the immunological perspective, immune cells infiltrate into adipose tissue, potentiating pro-inflammatory polarization and promoting the secretion of pro-inflammatory cytokines by leukocytes (29, 30). The long-term presence of these pro-inflammatory factors not only induces systemic inflammation but also gives rise to immune cell alterations at distal organ sites, leading to multi-organ dysfunction (31, 32). Obesity-related stress alters the immune environment at the maternal–fetal interface, disrupting normal placental function which can lead to pregnancy failure (33). Obesity can increase the risk of recurrent abortion. According to a systematic review and meta-analysis, women who experience recurrent pregnancy loss (RPL) have an average body mass index (BMI) that is considerably higher than that of the control group, indicating that maternal obesity could serve as a risk factor for RPL (34). In addition, being underweight seems to have a negative impact on in vitro fertilization (IVF) outcomes, but there are few conflicting studies assessing this phenomenon (35–37). The higher abortion rate in underweight women may be due to lower leptin levels (38), which may affect uterine angiogenesis and embryo implantation (39), impairing embryo implantation.

Smoking is a known risk factor for SA and can adversely affect pregnancy, embryonic development, and fetal health. Multiple studies have demonstrated that smokers have a higher risk of SA compared to non-smokers (39–41). Nicotine is a major addictive compound in tobacco smoke and a potent vasoconstrictor that reduces uterine and placental blood flow (42). Other toxic components of tobacco smoke include carbon monoxide, which binds to hemoglobin and reduces the oxygen supply to the fetus, and cyanide, which consumes vitamin B_12_, a necessary cofactor for fetal growth and development (43), thus having a negative impact on endometrial and embryonic development. Additionally, smoking may cause oxygen and nutrient deficiencies, leading to embryonic retardation and malformations. A comprehensive systematic review of 98 studies supports a significant association between active smoking behavior and an elevated risk of miscarriage. Moreover, this review revealed that the risk of miscarriage is further exacerbated when smoking exposure occurs during pregnancy, suggesting that smoking during pregnancy can have detrimental effects on fetal development and increase the likelihood of adverse pregnancy outcomes (41). A large cross-sectional study of 8,062 women found that smoking during reproductive years increased the risk of SA by 16%, stillbirth by 44%, and ectopic pregnancy by 43% compared to non-smokers (44). However, a case-control study of 620 women who had early abortions and 1,240 normal pregnant women reported no significant link between smoking fewer than 10 cigarettes and early abortion, despite a small percentage of women (3.1% to 3.4%) reporting smoking behavior (45). Recent research suggests that co-exposure to passive smoking and vitamin D deficiency may increase the risk of SA, with the risk increasing with a higher number of exposures (46). Therefore, smoking cessation and avoiding passive smoking exposure should be recommended as important strategies for reducing the risk of SA.

One of the key variables influencing SA is reproductive traits. AFB, AFS and AAM are important indicators of reproductive traits. In the present MR study, associations were observed between AFB, AFS, and late AAM and increased risk of SA later in life. The risk of abortion was the lowest in women aged 20-29, which was 12%, the risk of SA will increase in women who are too old or too young for the first birth (5). Younger women may have an immature reproductive system, which can increase the likelihood of complications during pregnancy. Additionally, younger women may be more prone to genetic abnormalities, which can lead to SA. Unhealthy behaviors such as smoking and alcohol consumption are known to increase the risk of miscarriage, and younger women may be more likely to engage in these activities. Finally, younger women may experience more stress due to factors such as lack of social support or financial difficulties, which have been linked to a higher risk of SA. The mechanism by which earlier sexual debut increases the risk of SA is not fully understood, but it may be related to physiological and behavioral factors. Adolescents who initiate sexual activity at a younger age are more likely to engage in risky sexual behaviors, such as having unprotected sex, having multiple sexual partners, and using drugs or alcohol. These behaviors may increase the risk of sexually transmitted infections (STIs), which are known to be associated with an increased risk of SA.

Studies have shown that women with early menarche (under 12 years old) and late menarche (14 years old or above) have more unsuccessful pregnancy outcomes than women with mid-menarche (12 or 13 years old) (47). A case-control study from Saudi Arabia showed that menarche age was positively correlated with SA risk (48). Another cross-sectional study of 3,743 women showed that late menarche was associated with being underweight and that menarche age was positively associated with final height (49). It is important to note that while the MR study provides evidence for a causal relationship between AAM and SA, it is not clear how strong this relationship is or how much of the risk is explained by other factors. More research is needed to fully understand the underlying mechanisms and to determine the clinical implications of these findings.

## Superiority and limitation

The use of a UVMR and MVMR design in our research is the key advantage of this investigation. This methodology enables us to analyze the possible causal connection between obesity, reproductive traits, smoking behaviors and SA based on large GWAS with an adequate sample size. The consistency between sensitivity analyses provides additional support for the efficacy of effect estimation. In addition, because SNPs are randomly distributed during conception, this design minimizes the confounding factors and reverse causality inherent in observational studies. And sensitivity analysis evaluates rigorously any violation of MR’s assumptions.

There are still potential limitations to our research. All relevant data come from studies that have analyzed Europeans separately. The homogeneity of the individuals eliminates the population stratification bias and provides accurate MR analysis results; moreover, it is yet to be determined whether or not our findings can be generalized to groups other than the ones we studied. Therefore, additional research involving additional populations is required to confirm the applicability of our results. Further research is required to research the causal relationship between obesity, smoking behaviors, reproductive traits, and SA.

## Conclusion

The positive causal relationship between AFS, waist circumference, leg fat percentage (left), smoking initiation, and SA was confirmed in this study, which is the first MR study to look into the connection between obesity, smoking behaviors, reproductive traits, and SA. Our findings demonstrate the significance of early intervention for pregnant women with obesity and smoking to prevent potential pregnancy complications. Moreover, our research helps with SA early prediction and risk stratification.

## Data availability statement

The original contributions presented in the study are included in the article/**Supplementary Material**. Further inquiries can be directed to the corresponding author.

## Author contributions

QW and LM: Conceptualization, Investigation, Writing – original draft. FL and YT: Investigation, Resources – original draft. XF: Project administration. All authors contributed to the article and approved the submitted version.

## Supplementary material

SUPPLEMENTARY FIGURE 1 Multivariable MR analysis results. 1: Multivariable MR-Egger method; 2: Multivariable MR-Lasso method; 3: Multivariable median method.
