# Supplementary figures and images for: Associations between obesity, smoking behaviors, reproductive traits and spontaneous abortion: a univariable and multivariable Mendelian randomization study
Source: Front Endocrinol (Lausanne). 2023 Jul 20;14:1193995. doi: 10.3389/fendo.2023.1193995 (PMC10400331; doi:10.3389/fendo.2023.1193995)

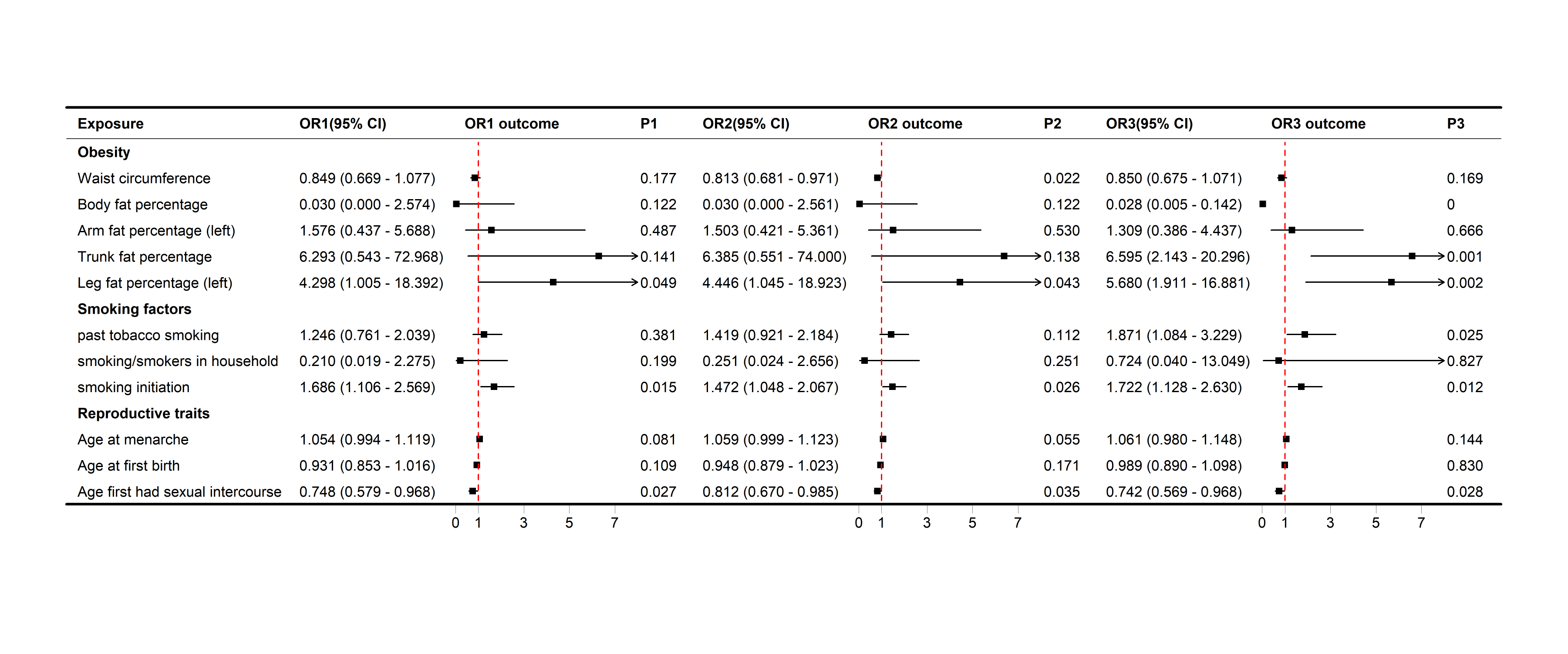

Supplement: SUPPLEMENTARY FIGURE 1 [file Image_1.tif]
